# Non-invasive diagnostic test for cow's milk allergy: A cross-sectional, descriptive study

**DOI:** 10.5339/qmj.2022.fqac.22

**Published:** 2022-04-04

**Authors:** Yosra Raziani, Brwa Othman, Asuda Ahmad, Sozyar Qadir

**Affiliations:** ^1^Department of Nursing, Komar University of Science and Technology, Sulaymaniyah, Kurdistan Region, Iraq E-mail: brwa.salahf19@komar.edu.iq

**Keywords:** CoMiSS, cow's milk allergy, non-invasive tests

## Abstract

Background: Cow's milk allergy is a common type of allergy in infants that is caused by the immune response to proteins in cow's milk. Digestive manifestations, atopic dermatitis, and respiratory discomfort are some of the clinical manifestations that appear; however, none of them are objective criteria to confirm the diagnosis, which may result in misdiagnosis, treatment hindrance, and parental concerns. Therefore, new methodologies for an accurate and immediate diagnosis is essential.

Methods: In this descriptive study, infants referred to the pediatric health center in Sulaymaniah were selected during a period of 1 year. The data were collected using a demographic questionnaire and the Cow's Milk Related Symptom Score (CoMiSS). Chi-squared and independent t tests were used to analyze the data.

Results: The findings of the present study indicated that among 250 infants (117 boys, 133 girls), with a mean ±  SD age of 2.9 ± 1.6 years, 21% were breastfed, 39% were fed both cow's milk and breast milk, and 60% were fed only cow's milk. The contingency of cow's milk allergy was positive in 35% of infants. According to this questionnaire, 18% of the participants got a score of 0–5, 47% scored 6–11, and 35% scored ≥ 12. A significant relationship was found between cow's milk allergy and the participants’ diet (p < 0.001). A significant association was also found between age-dependent growth index (weight p = 0.04, height p = 0.01, and head circumstance p = 0.02) and cow's milk allergy.

Conclusion: Although common problems in infancy such as colic and reflux may interfere with an accurate diagnosis of cow's milk allergy, give false-positive results, and decrease the reliability of CoMiSS; there is a need for non-invasive and easy methods for early diagnosis and improving awareness to encourage parents to take preventive measures.

